# Supplementary material for: Cationic crosslinked carbon dots-adjuvanted intranasal vaccine induces protective immunity against Omicron-included SARS-CoV-2 variants
Source: Nat Commun. 2023 May 9;14:2678. doi: 10.1038/s41467-023-38066-8 (PMC10169129; doi:10.1038/s41467-023-38066-8)
Supplement: Supplementary file 1 — Supplementary Information [file 41467_2023_38066_MOESM1_ESM.pdf]

Supplementary information

Cationic crosslinked carbon dot-adjuvanted intranasal vaccine induces protective immunity against Omicron-included SARS-CoV-2 variants

Contents:

Supplementary Text

Supplementary Fig. 1 to Fig. 14

Supplementary Text

Evaluation of the acid-base buffer capacity of CCD/RBD-HR

It was investigated *via* acid-base titration. Briefly, 5 mg of CCD was dissolved in 10 mL of 150 mM NaCl aqueous solution, and 1 N HCl was added to adjust the pH to 2.0. Then, 20 μL of 0.1 M NaOH was added, and the solution pH was measured with a pH meter (pHS-25) after each addition. For comparison, NaCl (150 mM) and PEI (1.8 kDa) were used under the same experimental conditions.

Isolation and culturing of NECs

Untreated, 6-8 weeks, female BALB/c mice were euthanized, and the nasal diaphragm mucosa was dissected. Briefly, the head skin was first dissected and completely removed to expose the skull, which was subsequently sectioned by scissors on the coronal section along two lines. The exposed septum was carefully acquired using surgical forceps, digested with 0.5% Type I collagenase, 0.5% Type IV collagenase, and 0.05% Trypsin in DMEM for 1 h at 37°C, filtered through 70 mesh cell strainers, and lysed with red blood cell lysis buffer to obtain single-cell suspensions. The cells were cultured in complete DMEM/F12 (Gibco) containing 10% FBS and 1% penicillin–streptomycin.

Investigation of the lysosome escaping capacity of CCD/RBD-HR

NECs were seeded in a 35 mm confocal dish at a density of 5×10^4 cells/well. After incubation for 24 h, the medium was removed, and 1 mL fresh culture medium containing CCD/RBD-HR (w/w= 10:1, 5 μg RBD-HR) was added to the dishes. RBD-HR was labeled with a fluorescent dye (green signal, Ex=488 nm). After incubation for 1, 2, 6, and 12 h, the lysosome (red signal, Ex=633 nm) and nucleus (blue signal, Ex=405 nm) of cells were stained with relative fluorescent dyes, and then cells were washed twice with 500 μL of PBS. The fluorescence images of the cells were visualized with an LSM 780 (Zeiss) confocal laser scanning microscope.

Cellular endocytosis pathway of CCD/RBD-HR

NECs were seeded in 48-well plates at a density of 5×10^4 cells. After 24 h, the cells were pretreated with low temperature (4 °C) or inhibition agents (cytochalasin D 5 μM, nocodazole 5 μM, genistein 40 μM and chlorpromazine 25 μM) for 30 min. RBD-HR was labeled with a 488-fluorescent dye. CCD/RBD-HR (w/w, 10:1, 0.4 μg RBD-HR per well) was added for 4 h, and then the cells were washed with 250 μL of PBS, harvested with 200 μL of 0.25% trypsin/EDTA, washed with 500 μL of PBS and resuspended in 150 μL of PBS. The fluorescence-positive cells were analyzed using a flow cytometer (Becton Dickinson and Company) with the FL3 channel (EX=488 nm).

Antibodies for flow cytometry

Antibodies were diluted at 1:100 in FCM assays. For DC and NEC activation, the following antibodies were obtained from BioLegend: FITC-conjugated anti-mouse CD80 (Cat. # 104706; Clone: 16-10A1), APC-conjugated anti-mouse CD40 (Cat. # 124612; Clone: 3/23), PE-conjugated anti-mouse CD86 (Cat. #159204; Clone: A17199A), PE/Cyanine7-conjugated anti-mouse MHC II (Cat. #107630; Clone: M5/114.15.2). For nasal epithelium–draining cervical lymph node (CLN) cell recruitment, the following antibodies were obtained from BioLegend: FITC-conjugated anti-mouse CD11b (Cat. #101206; Clone: M1/70), APC-conjugated anti-mouse CD11c (Cat. #117310; Clone: N418), PerCP/Cyanine5.5-conjugated anti-mouse F4/80 (Cat. #123128; Clone: BM8), Brilliant Violet 421-conjugated anti-mouse ly6C (Cat. #128032; Clone: HK1.4), Brilliant Violet 510-conjugated anti-mouse ly6G (Cat. #127633; Clone: 1A8) and Brilliant Violet 711-conjugated anti-mouse MHC II (Cat. #107643; Clone: M5/114.15.2). For germinal center (GC) B cells, the following antibodies were obtained from BioLegend: PE-conjugated anti-mouse CD3 (Cat. #100206; Clone: 17A2), PerCP/Cyanine5.5-conjugated anti-mouse CD19 (Cat. #152406; Clone: 1D3/CD19), FITC-conjugated anti-mouse CD95 (Cat. #152606; Clone: SA367H8), APC-conjugated anti-mouse GL-7 (Cat. # 144618; Clone: GL7). For Tfh cell staining, the following antibodies were used: PE-conjugated anti-mouse CD4 (Cat. #100206; Clone: 17A2), FITC-conjugated anti-mouse CD279 (PD-1, Cat. #135214; Clone: 29F.1A12) and APC-conjugated anti-mouse185 (CXCR5, Cat. #145506; Clone: L138D7). For cell recruitment to BAL fluid, cells were stained with FITC-conjugated anti-mouse CD11b (Cat. #101206; Clone: M1/70), APC-conjugated anti-mouse CD11c (Cat. #117310; clone: N418), Brilliant Violet 711-conjugated anti-mouse MHC II (Cat. #107643; Clone: M5/114.15.2), and Brilliant Violet 510-conjugated anti-mouse LY6G (Cat. #127633; Clone: 1A8). For the detection of T_{RM}s in BALs and lungs, the following antibodies were used: PerCP/Cyanine5.5-conjugated anti-mouse CD3 (Cat. 100218; Clone: 17A2), Brilliant Violet 421-conjugated anti-mouse CD4 (Cat. #100438; Clone: GK1.5), FITC-conjugated anti-mouse CD8a (Cat. #100804, Clone: 5H10-1), Brilliant Violet 510-conjugated anti-mouse CD44 (Cat. # 103044, Clone: IM7), PE-conjugated anti-mouse CD69 (Cat. # 104508; Clone: H1.2F3), APC-conjugated anti-mouse CD103 (Cat. # 121414, Clone: 2E7). For the detection of CD103$^+$ DCs, the following antibodies were obtained from BioLegend: FITC-conjugated anti-mouse CD11b (Cat. #101206; Clone: M1/70), PE/Cyanine7-conjugated anti-mouse CD11c (Cat. #117318; clone: N418), APC-conjugated anti-mouse CD103 (Cat. # 121414, Clone: 2E7) and PE-conjugated anti-mouse CD86 (Cat. #159204; Clone: A17199A). Functional T cells in the lungs were stained with the following antibodies: PerCP/Cyanine 5.5-conjugated anti-mouse CD3 (Cat. 100218; Clone: 17A2), APC-conjugated anti-mouse CD4 (Cat. #100412; Clone: GK1.5), FITC-conjugated anti-mouse CD8a (Cat.

#100804, Clone: 5H10-1), PE-conjugated anti-mouse IFN-γ (Cat. #505808; Clone: XMG1.2) and Brilliant Violet 421 conjugated anti-mouse TNF-α (Cat. # 506328; Clone: MP6-XT22). OVA-specific CD4 T^{+} cells were stained with FITC-conjugated anti-mouse CD4 (Cat. #100406; Clone: GK1.5) and PE-$OVA_{323-339}$ tetramer (TS-M710-1, MBL); OVA-specific $CD8^{+}$ T cells were stained with Brilliant Violet 510-conjugated anti-mouse CD8a (Cat. #100804, Clone: 5H10-1) and PE-$OVA_{257-264}$ tetramer (TS-5001-1C, MBL). For splenic T cells, the following antibodies were obtained from BioLegend: PerCP/Cyanine5.5-conjugated anti-mouse CD3 (Cat. #100218; clone: 17A2), APC-conjugated anti-mouse CD4 (Cat. #100412; Clone: GK1.5), FITC-conjugated anti-mouse CD8a (Cat. #100804, Clone: 5H10-1), Brilliant Violet 421-conjugated anti-mouse CD44 (Cat. # 103044, Clone: IM7) and PE-conjugated anti-mouse CD69 (Cat. #104508; Clone: H1.2F3). IgG-producing plasma cells in bone marrow and blood were stained with APC-conjugated anti-mouse IgG (Cat. #405308; Clone: Poly4053), PerCP/Cyanine5.5-conjugated anti-mouse B220 (Cat. #152406; Clone: 1D3/CD19), Brilliant Violet 510 anti-mouse IgD (Cat. #405723; Clone: 11-26c, 2a), and Brilliant Violet 421 anti-mouse CD138 (Cat. #142508; Clone: 281-2). PE/Cyanine7 anti-mouse CD38 (Cat. #102718; Clone: 90) and FITC-conjugated anti-mouse CD80 (Cat. #104706; Clone: 16-10A1) were used to stain memory B cells in spleen, bone marrow, and blood. Functional T cells in the spleen were stained with the following antibodies: PerCP/Cyanine 5.5-conjugated anti-mouse CD3 (Cat. 100218; Clone: 17A2), PE-conjugated anti-mouse CD4 (Cat. #100206; Clone: 17A2), FITC-conjugated anti-mouse CD8a (Cat. #100804, Clone: 5H10-1), APC-conjugated anti-mouse IL-4 (Cat. #504105; Clone: 11B11) and PE/Cyanine7-conjugated anti-mouse IFN-γ (Cat. #505808; Clone: XMG1.2).

Supplementary Fig. 1

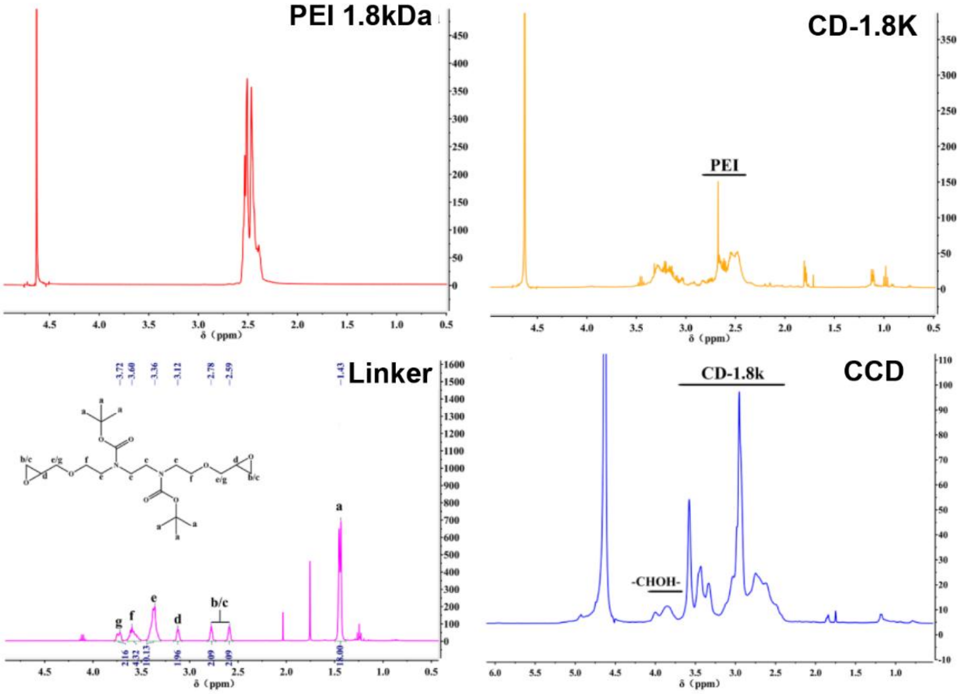

Supplementary Fig. 1 Chemical structures of PEI 1.8 kDa, CD-1.8k, Linker, and CCD analyzed by 1H-NMR.

Supplementary Fig. 2

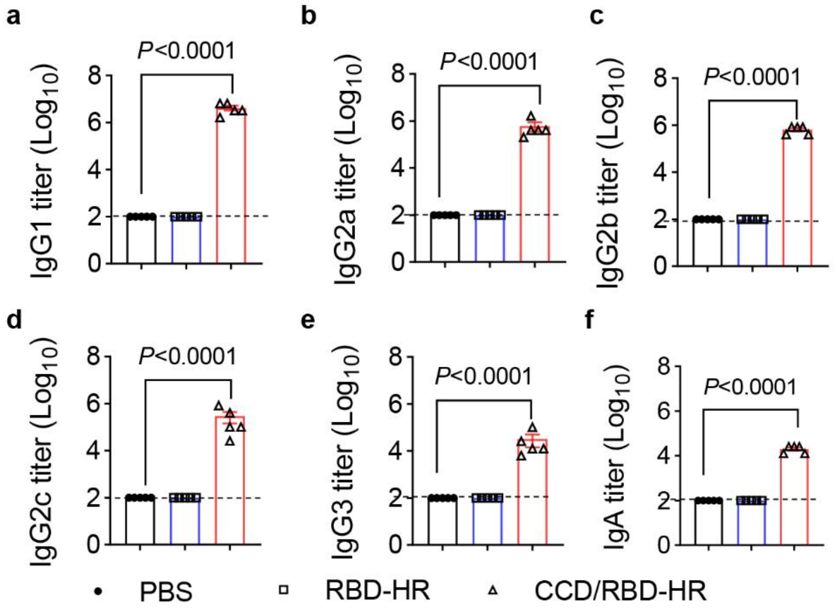

Supplementary Fig. 2 IgG isotypes and IgA analysis. BALB/c mice (*n* = 5 per group) were immunized intranasally with 10 μg RBD-HR, alone or with 100 μg CCD, on days 0, 14, and 28. On day 35, the immune sera were collected for the detection of IgG1 (**a**), IgG2a (**b**), IgG2b (**c**), IgG2c (**d**), IgG3 (**e**), and IgA (**f**) with ELISA. Data were presented as mean values ± SEM. *P* values were calculated with One-way ANOVA followed by Dunnett's multiple comparisons test. Source data are provided as a Source Data file.

Supplementary Fig. 3

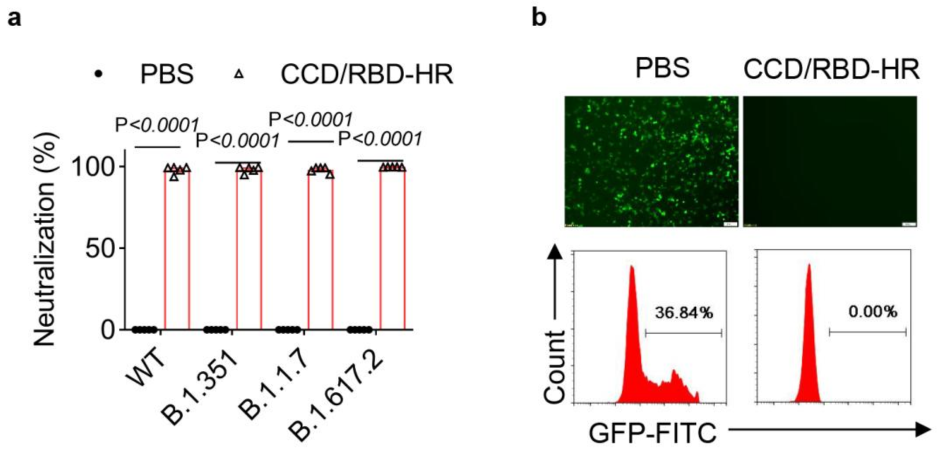

Supplementary Fig. 3 Functional characterization of the induced antibodies. Mouse immune sera were collected 35 days after the first immunization. **a** Inhibition of WT or mutated RBD binding to ACE2 receptors by serum antibodies was evaluated with FCM at a 1:270 dilution. **b** Serum diluted 1:270 neutralized EGFP-expressing SARS-CoV-2 pseudovirus infection in 293T/ACE2 cells, as determined by FCM and fluorescence microscopy. *n* = 5 mice per group. Data were presented as mean values ± SEM in **a**. Source data are provided as a Source Data file.

Supplementary Fig. 4

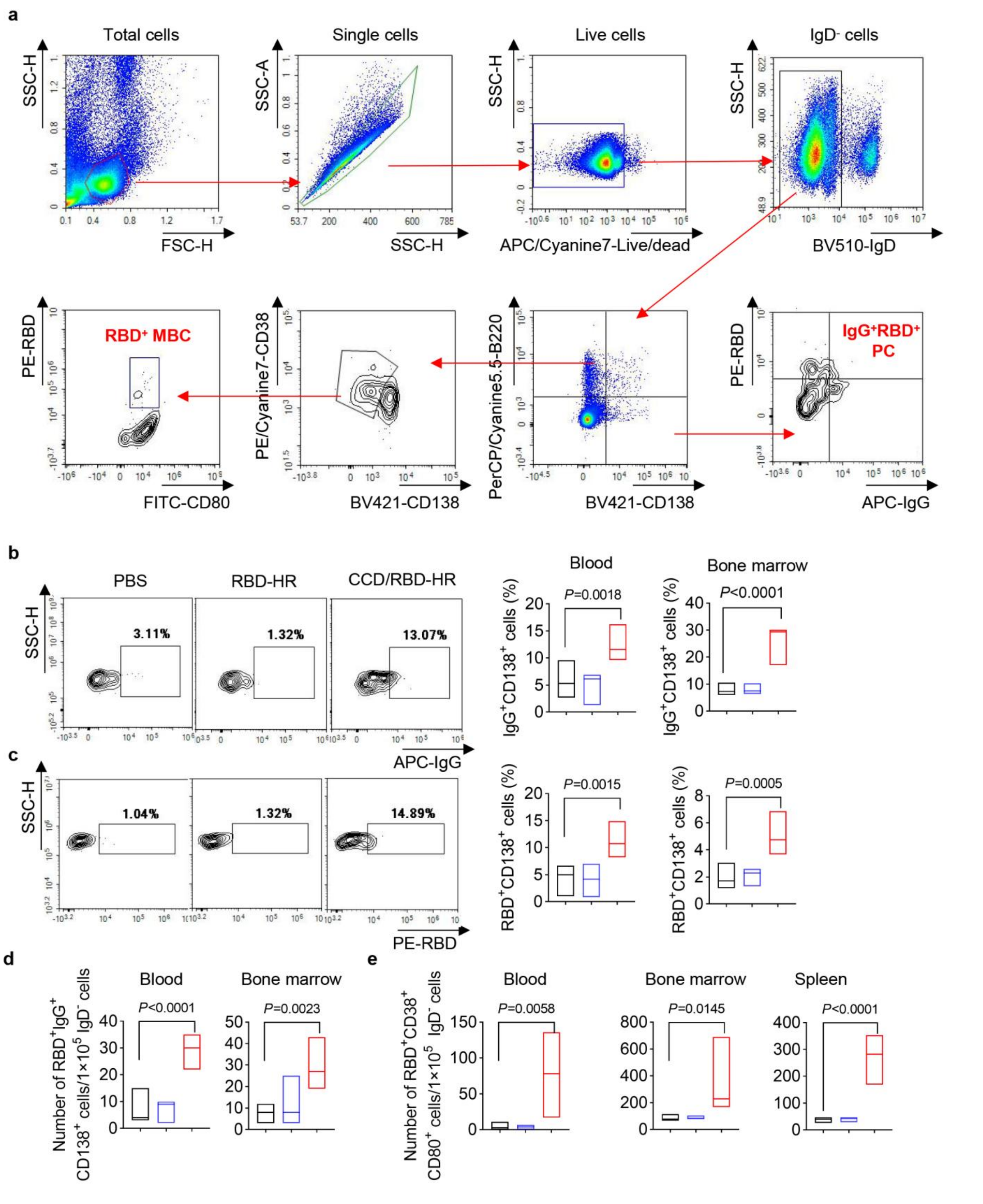

Supplementary Fig. 4 B-cell responses four weeks after the last CCD/RBD-HR immunization.

a Gating strategy for the measurement of RBD-specific IgG-producing plasma cells ($IgD^-B220^-CD138^+IgG^+RBD^+$) and MBC ($IgD^-CD138^-B220^+CD38^+CD80^+RBD^+$) responses. **b**, **c**

Representative FCM plots (left) and quantification (middle and right) of IgG-producing (**b**) and RBD-specific (**c**) $CD138^+$ plasma cells in blood and bone marrow. The number of RBD-specific IgG-producing $CD138^+$ plasma cells in the blood and bone marrow (**d**), memory B cells ($RBD^+CD38^+CD80^+$) in the blood, bone marrow, and spleen (**e**) were assayed with FCM. Data are displayed with floating bars in **b**-**e**. The middle line indicates the median, and the box shows the data range. Data were presented as mean values ± SEM. $n = 5$ mice per group. P values in **b**-**e** were calculated with One-way ANOVA followed by Dunnett's multiple comparisons test. Source data are provided as a Source Data file.

Supplementary Fig. 5

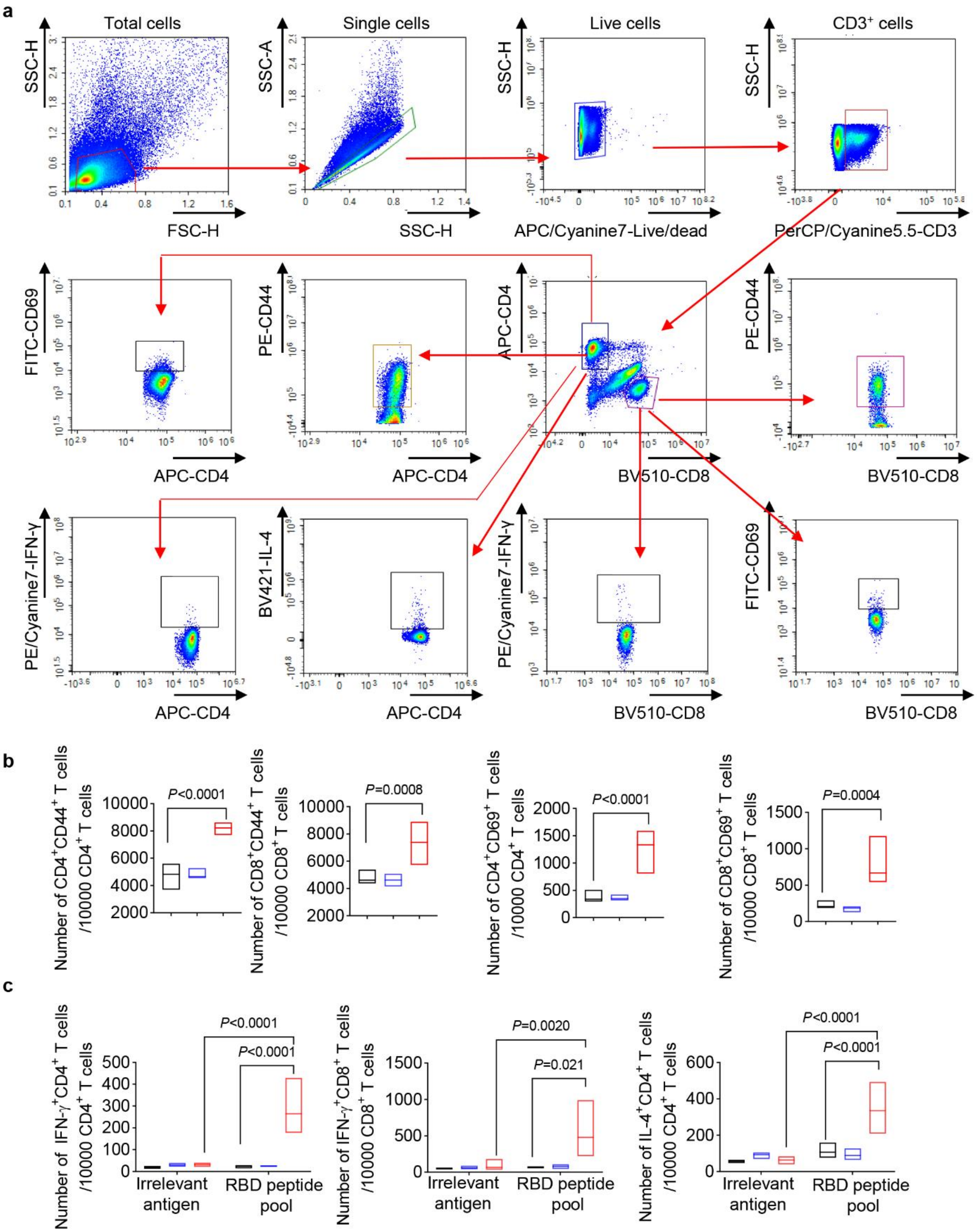

Supplementary Fig. 5 T-cell responses in spleen four weeks after the last CCD/RBD-HR immunization. a, Gating for the detection of splenic T-cell responses four weeks after the last

immunization. **b** The number of antigen-experienced (CD44$^+$) and activated (CD69$^+$) CD4$^+$/CD8 T$^+$ cells in the spleen were determined with FCM after re-stimulation with RBD protein peptide pools. **c** The number of IL-4 and IFN-γ expressed CD4$^+$/CD8 T$^+$ cells in the spleen were determined with intracellular cytokine staining after re-stimulation with RBD protein peptide pools. Data are displayed with floating bars in **b** and **c**. The middle line indicates the median, and the box shows the data range. Data were presented as mean values ± SEM. *n* = 5 mice per group. *P* values in **b** and **c** were calculated with One-way ANOVA followed by Dunnett's multiple comparisons test. Source data are provided as a Source Data file.

Supplementary Fig. 6

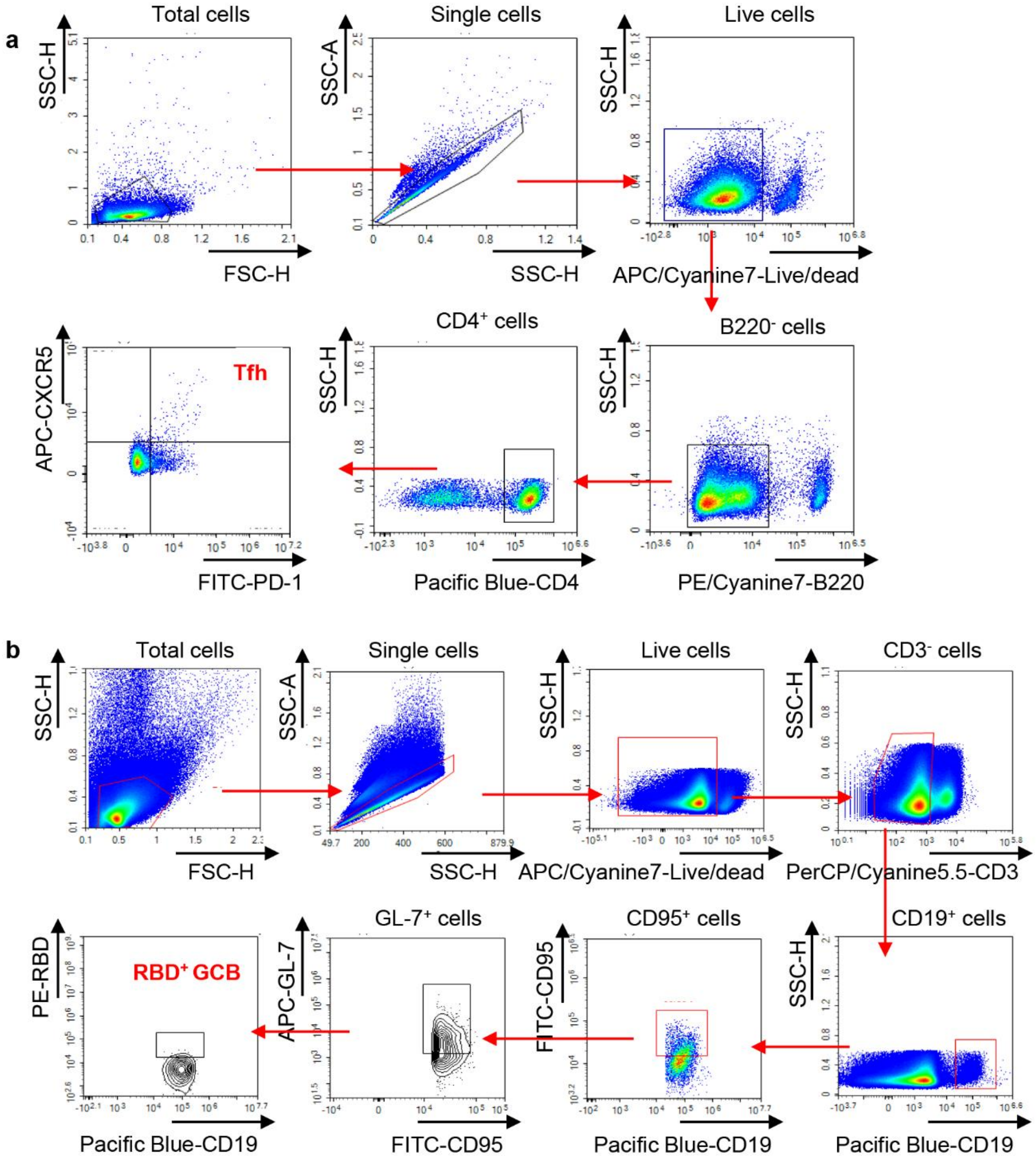

Supplementary Fig. 6 Gating for the detection of Tfh (a) and RBD-specific GCB responses (b) in the draining CLN four weeks after the last immunization.

Supplementary Fig. 7

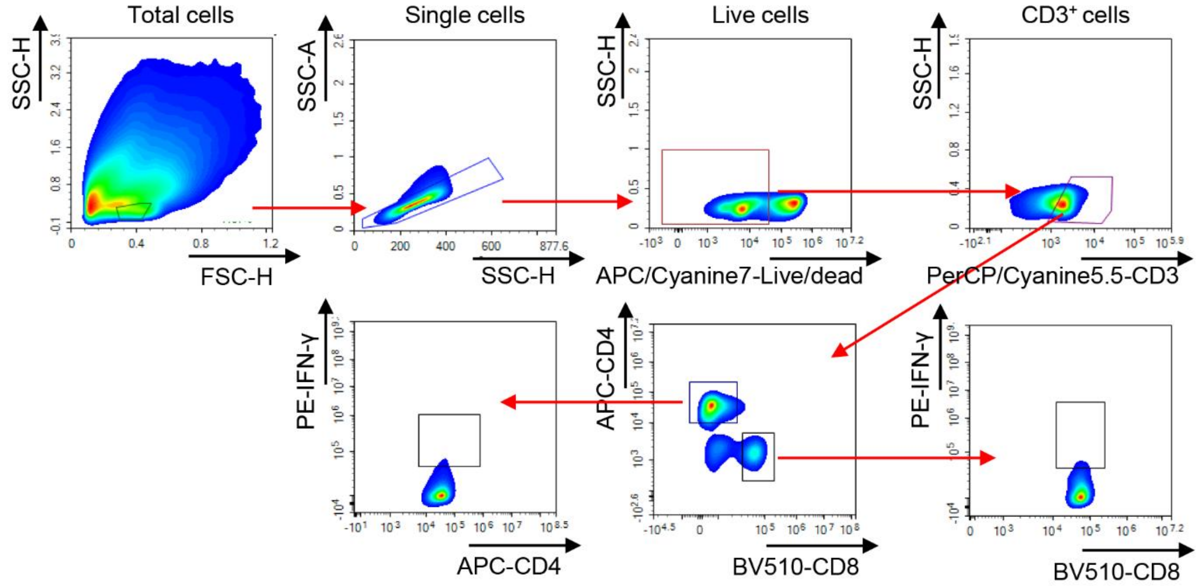

Supplementary Fig. 7 Gating for the detection of lung T-cell responses four weeks after the last immunization.

Supplementary Fig. 8

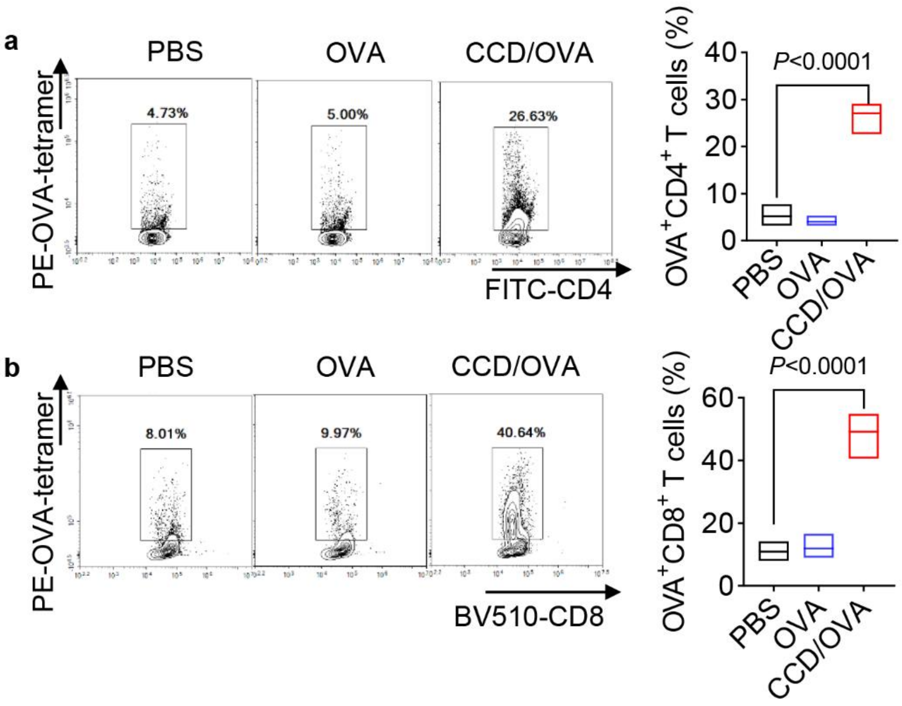

Supplementary Fig. 8 OVA-specific T-cell responses in the lungs. C57BL/6 mice (n = 5 per group) were intranasally immunized with 10 μg OVA with or without 100 μg CCD on days 0, 14, and 28. On day 35, the generation of antigen-specific $CD4^+$ (**a**) and CD8 T^+ (**b**) cell responses in the lungs were determined by FCM with I-A^d-restricted $OVA_{323–339}$ tetramer and H-$2K^b$-restricted $OVA_{257–264}$ (SIINFEKL), respectively. Representative FCM plots (left) and quantification results (right) were displayed. Data are displayed with floating bars. The middle line indicates the median, and the box shows the data range. Data were presented as mean values ± SEM. *P* values were calculated with One-way ANOVA followed by Dunnett's multiple comparisons test. Source data are provided as a Source Data file.

Supplementary Fig. 9

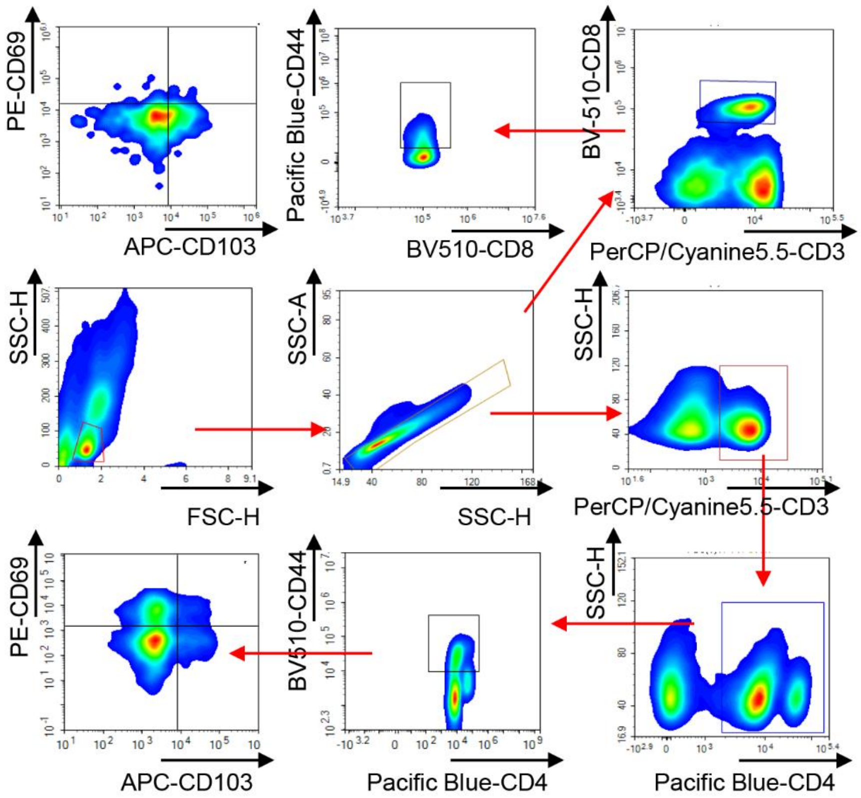

Supplementary Fig. 9 RBD-specific T cell responses in the lungs four weeks after the last CCD/RBD-HR immunization. Gating strategy for the measurement of IFN-γ-producing $CD4^+$ and $CD8^+$ T cells in the lungs at four weeks after the last boost.

Supplementary Fig. 10

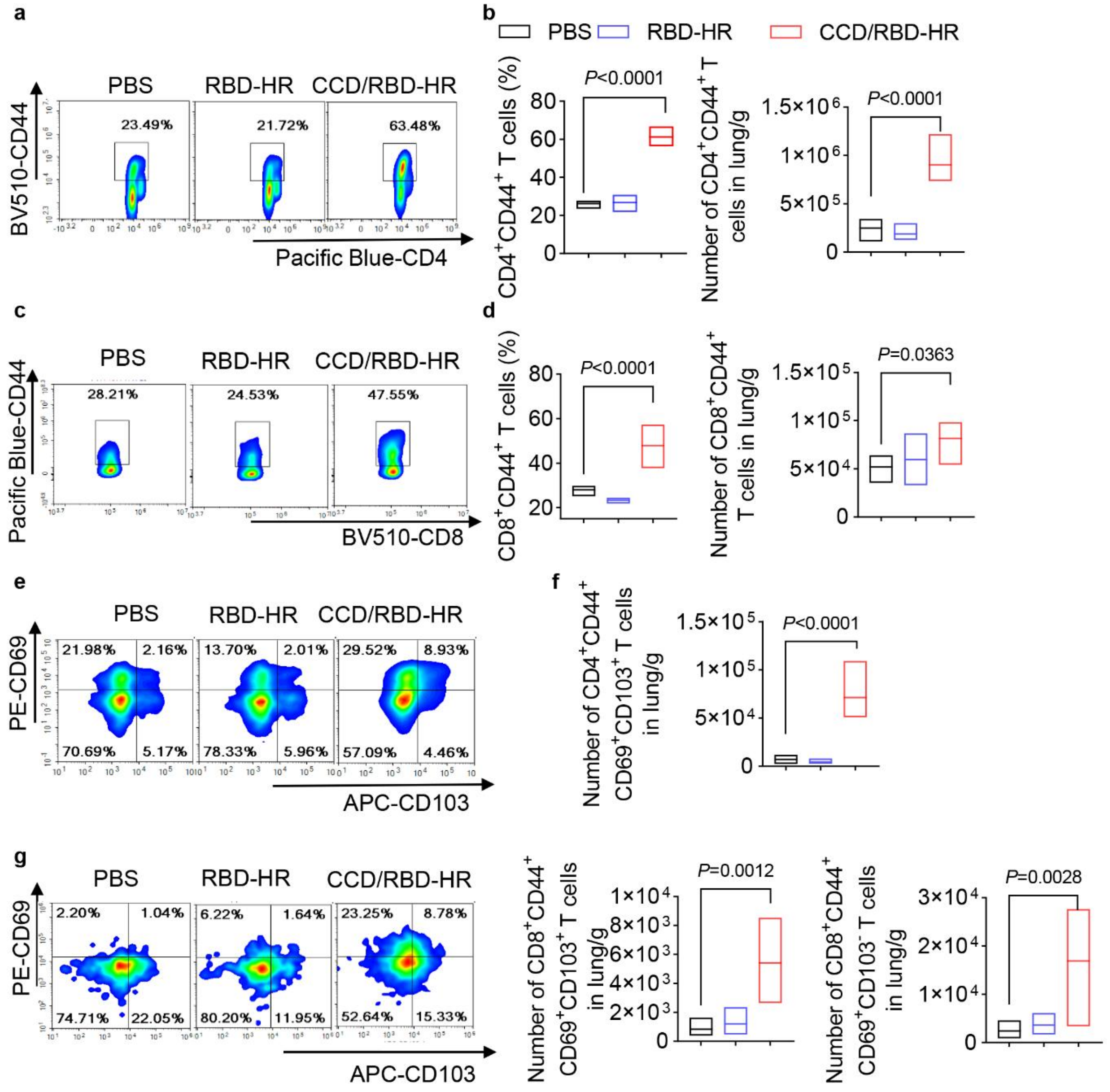

Supplementary Fig. 10 T-cell responses in the lungs four weeks after the last CCD/RBD-HR immunization. **a** FCM plots of $CD4^{+}CD44^{+}$ T cells in the lungs. **b** Quantification of the percentages (left) and the numbers (right) of $CD4^{+}CD44^{+}$ T cells in the lungs. **c** FCM plots of $CD8^{+}CD44^{+}$ T cells in the lungs. **d** Quantification of the percentages (left) and the numbers (right) of $CD8^{+}CD44^{+}$ T cells in the lungs. **e** FCM plots of $CD4^{+}CD44^{+}$ cells expressing CD69, CD103, or both. **f** Quantification of the numbers of $CD4^{+}CD44^{+}CD69^{+}CD103^{+}$ T_{RM} cells in the lungs. **g** FCM plots of $CD8^{+}CD44^{+}$ cells expressing CD69, CD103, or both. **h** Quantification of the numbers of $CD8^{+}CD44^{+}CD69^{+}CD103^{+}$ (left) and $CD8^{+}CD44^{+}CD69^{+}CD103^{-}$ (right) T_{RM} cells in the lungs. Data are displayed with floating bars in **b**, **d**, **f**, and **g**. The middle line indicates the

median, and the box shows the data range. Data were presented as mean values ± SEM. n = 5 mice per group. P values were calculated with One-way ANOVA followed by Dunnett's multiple comparisons test. Source data are provided as a Source Data file.

Supplementary Fig. 11

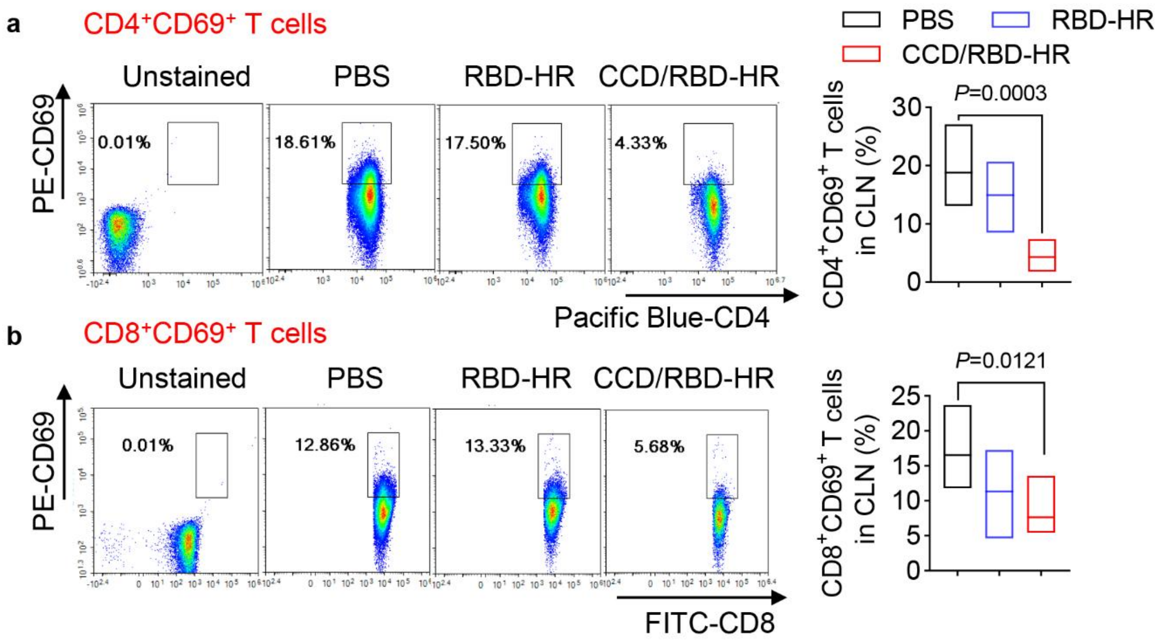

Supplementary Fig. 11 T-cell responses in the CLN one week after the last CCD/RBD-HR immunization. FCM dot plots (left) and quantification of the fractions of (right) $CD4^+CD69^+$ (**a**) and $CD8^+CD69^+$ T (**b**) cells in the CLN. Data are displayed with floating bars. The middle line indicates the median, and the box shows the data range. Data were presented as mean values ± SEM. *n* = 5 mice per group. *P* values were calculated with One-way ANOVA followed by Dunnett's multiple comparisons test. Source data are provided as a Source Data file.

Supplementary Fig. 12

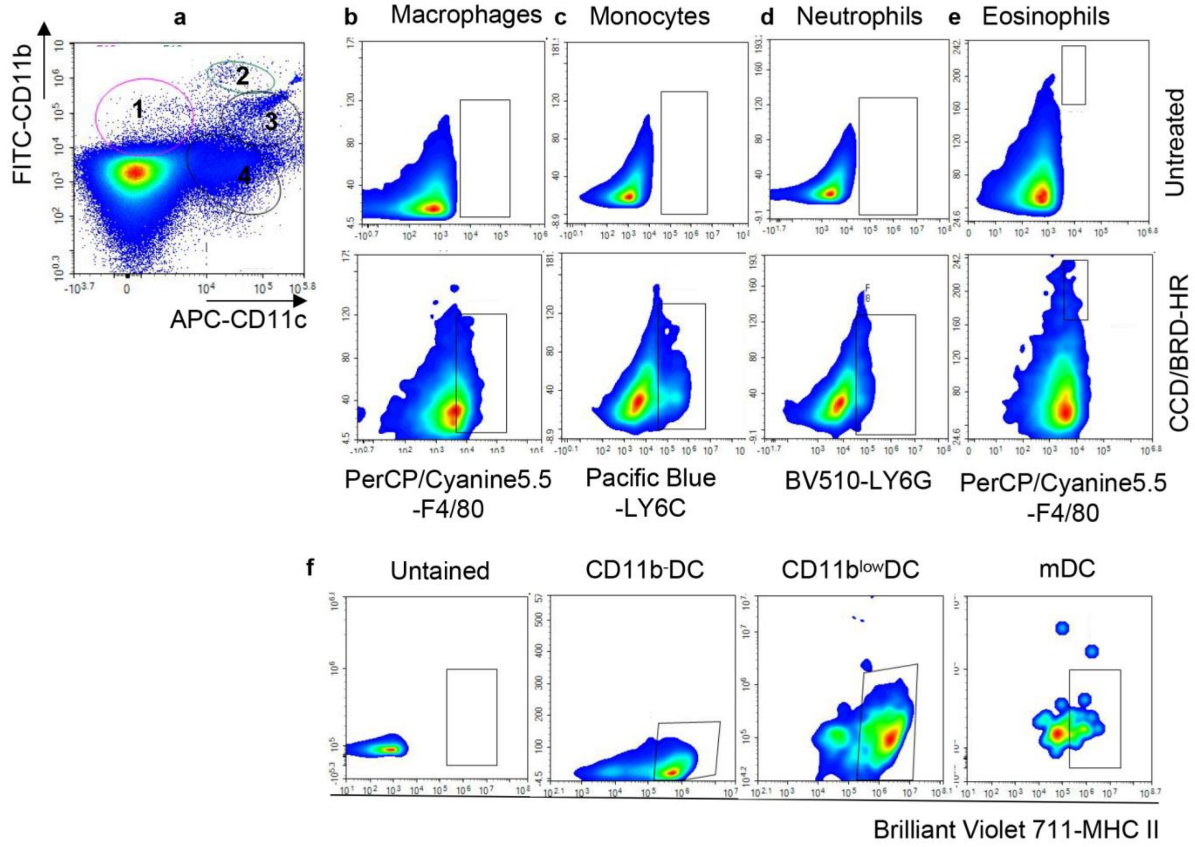

Supplementary Fig. 12 Gating for the recruitment of different immune cells in the draining CLN. a Dot plot of CLN cells collected 72 h after intranasal CCD/RBD-HR vaccine inoculation. The x-y axis refers to CD11c-APC and CD11b-FITC. Region 1 includes macrophages, infiltrating monocytes, neutrophils, and eosinophils. Region 2 represents mDCs, region 3 represents $CD11b^{low}$ DCs and region 4 represents $CD11b^{-}$ DCs. The gating for (**b**) macrophages, (**c**) monocytes, (**d**) neutrophils, and (**e**) eosinophils is shown. (**f**) Left to right represents the gating for unstained, $CD11b^{-}$ DCs, $CD11b^{blow}$ DCs, and mDCs. Representative plots are shown from experiments with n = 5 mice.

Supplementary Fig. 13

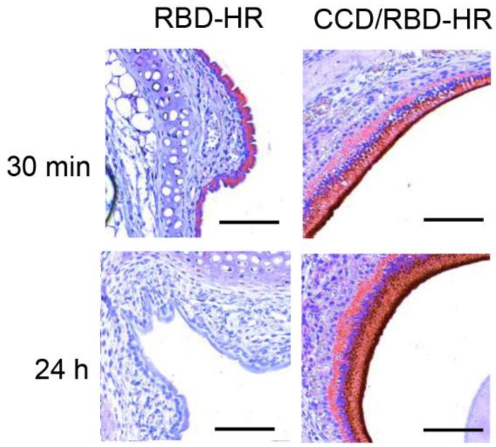

Supplementary Fig. 13 Binding of RBD-HR to the nasal mucosa after immunization with CCD/RBD-HR. BALB/c mice (*n* = 3 per group) were intranasally immunized with RBD-HR or CCD/RBD-HR. 30 min and 24 h later, the mice were sacrificed, and the nasal mucosa was harvested to determine the RBD-HR residue at the mucosal sites of the nasal cavity with immunohistochemical analysis. Scale bar, 100 μm.

Supplementary Fig. 14

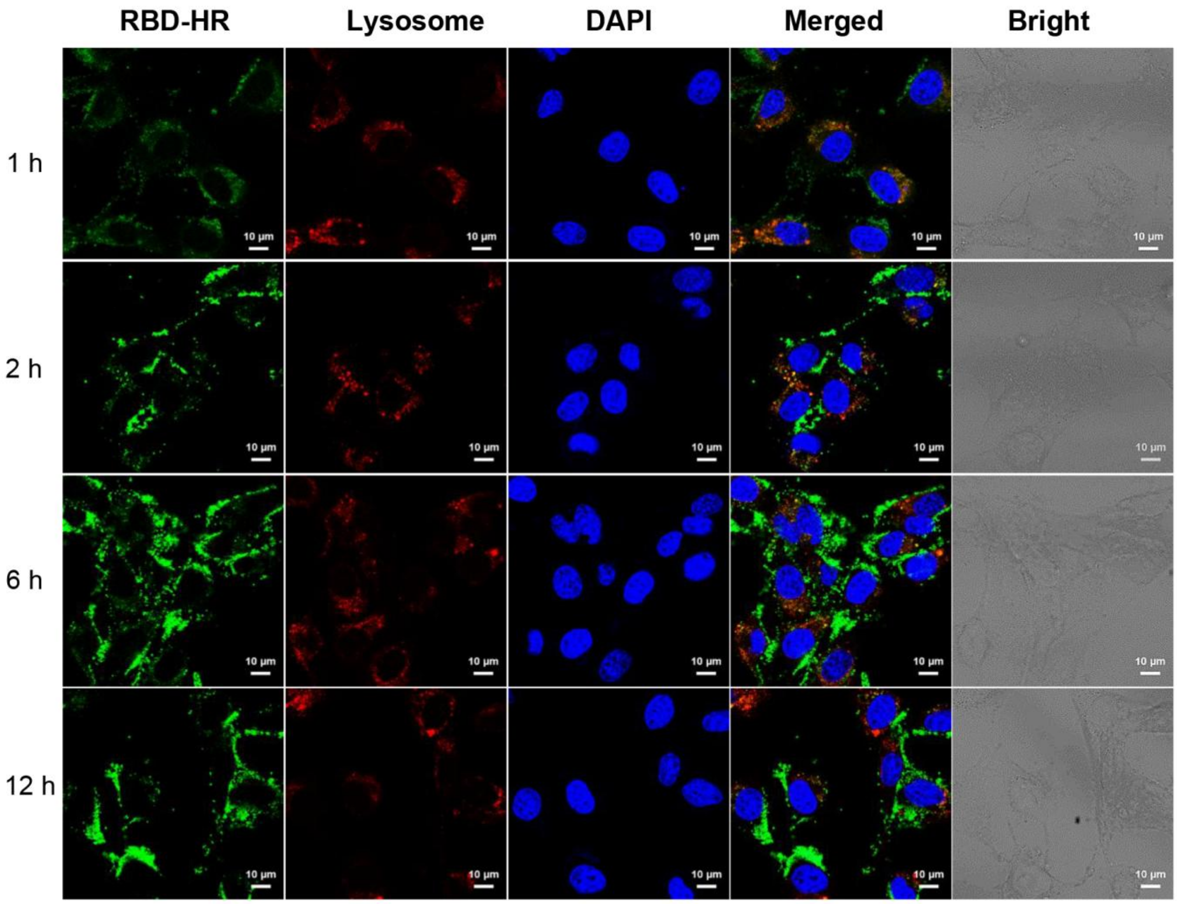

Supplementary Fig. 14 The lysosome process of CCD/RBD-HR. After NECs were seeded and cultured for 24 h, the medium was removed and replaced with 1 mL fresh culture medium containing the CCD/fluorescent dye-labeled RBD-HR (w/w, 10:1, 5 μg RBD-HR). After incubation for 1, 2, 6, and 12 h, the lysosome (red signal, Ex=633 nm) and nucleus (blue signal, Ex=405 nm) of cells were stained with relative fluorescent dyes, and then cells were washed twice with 500 μL PBS. The fluorescence images of the cells were visualized with an LSM 780 (Zeiss) confocal laser scanning microscope. Similar results were obtained in three independent experiments.
