# Supplementary material for: Cationic crosslinked carbon dots-adjuvanted intranasal vaccine induces protective immunity against Omicron-included SARS-CoV-2 variants
Source: Nat Commun. 2023 May 9;14:2678. doi: 10.1038/s41467-023-38066-8 (PMC10169129; doi:10.1038/s41467-023-38066-8)
Supplement: Supplementary file 3 — Reporting Summary [file 41467_2023_38066_MOESM3_ESM.pdf]

nature portfolio

Corresponding author(s): Xiawei Wei

Last updated by author(s): April 10, 2023

Reporting Summary

Nature Portfolio wishes to improve the reproducibility of the work that we publish. This form provides structure for consistency and transparency in reporting. For further information on Nature Portfolio policies, see our Editorial Policies and the Editorial Policy Checklist.

Statistics

For all statistical analyses, confirm that the following items are present in the figure legend, table legend, main text, or Methods section.

n/a	Confirmed	
☐	☒	The exact sample size (*n*) for each experimental group/condition, given as a discrete number and unit of measurement
☐	☒	A statement on whether measurements were taken from distinct samples or whether the same sample was measured repeatedly
☐	☒	The statistical test(s) used AND whether they are one- or two-sided<br>*Only common tests should be described solely by name; describe more complex techniques in the Methods section.*
☒	☐	A description of all covariates tested
☐	☒	A description of any assumptions or corrections, such as tests of normality and adjustment for multiple comparisons
☐	☒	A full description of the statistical parameters including central tendency (e.g. means) or other basic estimates (e.g. regression coefficient) AND variation (e.g. standard deviation) or associated estimates of uncertainty (e.g. confidence intervals)
☐	☒	For null hypothesis testing, the test statistic (e.g. *F*, *t*, *r*) with confidence intervals, effect sizes, degrees of freedom and *P* value noted<br>*Give P values as exact values whenever suitable.*
☒	☐	For Bayesian analysis, information on the choice of priors and Markov chain Monte Carlo settings
☒	☐	For hierarchical and complex designs, identification of the appropriate level for tests and full reporting of outcomes
☒	☐	Estimates of effect sizes (e.g. Cohen's *d*, Pearson's *r*), indicating how they were calculated

Our web collection on statistics for biologists contains articles on many of the points above.

Software and code

Policy information about availability of computer code

Data collection

1. Flow cytometric data were collected using NovoCyte with NovoExpress 1.4.1 (ACEA bioscience. Inc).
2. Firefly luciferase assay data were collected using multi-mode microplate reader with kaleido 3.0 (PerkinElmer).
3. Fluorescent images were obtained using Olympus IX73 microscope with CellSens Standard software 2.1 (Olympus Corporation).
4. Immunofluorescence and immunohistochemistry were obtained using fluorescence microscope with leica LAS X software (Leica, Germany).
5. Enzyme linked Immunosorbent assay data were collected using IRIS FluoroSpot/ELISpot reader (Mabtech).
6. Pathologic slides were digitized using Pannoramic MIDI with scanning software:Pannoramic Scanner and browsing software:Caseviewer : C.V 2.3 (3DHISTECH)
7. The fluorescence images of the cells for lysosomal escape were visualized using an LSM 780 confocal laser scanning microscope with ZEN software (Zeiss) .

Data analysis

The statistical analyses were performed with GraphPad Prism version 8.0 for One- or Two-way ANOVA test. Flow cytometry data were analyzed by FlowJo V.10 software and NovoExpress 1.4.1.

For manuscripts utilizing custom algorithms or software that are central to the research but not yet described in published literature, software must be made available to editors and reviewers. We strongly encourage code deposition in a community repository (e.g. GitHub). See the Nature Portfolio guidelines for submitting code & software for further information.

Data

Policy information about availability of data

All manuscripts must include a data availability statement. This statement should provide the following information, where applicable:

- Accession codes, unique identifiers, or web links for publicly available datasets
- A description of any restrictions on data availability
- For clinical datasets or third party data, please ensure that the statement adheres to our policy

All data that support the findings of this study are available with the paper and its Supplementary information. Source data are provided with this paper.

Human research participants

Policy information about studies involving human research participants and Sex and Gender in Research.

Reporting on sex and gender	N/A
Population characteristics	N/A
Recruitment	N/A
Ethics oversight	N/A

Note that full information on the approval of the study protocol must also be provided in the manuscript.

Field-specific reporting

Please select the one below that is the best fit for your research. If you are not sure, read the appropriate sections before making your selection.

☒ Life sciences ☐ Behavioural & social sciences ☐ Ecological, evolutionary & environmental sciences

For a reference copy of the document with all sections, see nature.com/documents/nr-reporting-summary-flat.pdf

Life sciences study design

All studies must disclose on these points even when the disclosure is negative.

Sample size	Power test was performed with known mean values and SD that are related to this study. The type I error was set at 0.05 and desired power at 0.80.
Data exclusions	No data were excluded from the analysis.
Replication	Most of experiments were repeated with at least three biological and technical triplicates for all results presented in the manuscript.
Randomization	In the animal studies, mice and rabbits were randomly assigned to different treatment groups.
Blinding	For animal immunization, technicians were not blinded to group allocation as they need to know the identity document of the corresponding vaccinated animal. For date collection and analysis of other in vivo and in vitro experiment, the investigators were blinded to group allocations.

Reporting for specific materials, systems and methods

We require information from authors about some types of materials, experimental systems and methods used in many studies. Here, indicate whether each material, system or method listed is relevant to your study. If you are not sure if a list item applies to your research, read the appropriate section before selecting a response.

Materials & experimental systems

n/a	Involved in the study	
☐	☒	Antibodies
☐	☒	Eukaryotic cell lines
☒	☐	Palaeontology and archaeology
☐	☒	Animals and other organisms
☒	☐	Clinical data
☒	☐	Dual use research of concern

Methods

n/a	Involved in the study	
☒	☐	ChIP-seq
☐	☒	Flow cytometry
☒	☐	MRI-based neuroimaging

Antibodies

Antibodies used

The following antibodies were used at 1:100 dilution for flow cytometry.
FITC-conjugated anti-mouse CD80 antibody: Provide supplier name: BioLegend, Cat. # 104706, Clone: 16-10A1.
APC-conjugated anti-mouse CD40 antibody: Provide supplier name: BioLegend, Cat. # 124612, Clone: 3/23.
PE-conjugated anti-mouse CD86 antibody: Provide supplier name: BioLegend, Cat. #159204, Clone: A17199A.
PE/Cyanine7-conjugated anti-mouse MHC II antibody: Provide supplier name: BioLegend, Cat. #107630, Clone: M5/114.15.2.
FITC-conjugated anti-mouse CD11b antibody: Provide supplier name: BioLegend, Cat. #101206, Clone: M1/70.
APC-conjugated anti-mouse CD11c antibody: Provide supplier name: BioLegend, Cat. #117310, Clone: N418.
PerCP/Cyanine5.5-conjugated anti-mouse F4/80 antibody: Provide supplier name: BioLegend, Cat. #123128, Clone: BM8.
Brilliant Violet 421-conjugated anti-mouse ly6C antibody: Provide supplier name: BioLegend, Cat. #128032, Clone: HK1.4.
Brilliant Violet 510-conjugated anti-mouse ly6G antibody: Provide supplier name: BioLegend, Cat. #127633, Clone: 1A8.
Brilliant Violet 711-conjugated anti-mouse MHC II antibody: Provide supplier name: BioLegend, Cat. #107643, Clone: M5/114.15.2.
PE-conjugated anti-mouse CD3 antibody:Provide supplier name: BioLegend, Cat. #100206, Clone: 17A2.
PerCP/Cyanine5.5-conjugated anti-mouse CD19 antibody:Provide supplier name: BioLegend, Cat. #152406, Clone: 1D3/CD19.
FITC-conjugated anti-mouse CD95 antibody:Provide supplier name: BioLegend, Cat. #152606, Clone: SA367H8.
APC-conjugated anti-mouse GL-7 antibody:Provide supplier name: BioLegend, Cat. # 144618, Clone: GL7.
PE-conjugated anti-mouse CD4 antibody:Provide supplier name: BioLegend, Cat. #100206, Clone: 17A2.
FITC-conjugated anti-mouse CD279 (PD-1) antibody:Provide supplier name: BioLegend, Cat. #135214, Clone: 29F.1A12.
APC-conjugated anti-mouse185 (CXCR5) antibody:Provide supplier name: BioLegend, Cat. #145506, Clone: L138D7.
PerCP/Cyanine5.5-conjugated anti-mouse CD3 antibody:Provide supplier name: BioLegend, Cat. 100218, Clone: 17A2.
Brilliant Violet 421-conjugated anti-mouse CD4 antibody:Provide supplier name: BioLegend, Cat. #100438, Clone: GK1.5.
FITC-conjugated anti-mouse CD8a antibody: Provide supplier name: BioLegend, Cat. #100804, Clone: 5H10-1.
Brilliant Violet 510-conjugated anti-mouse CD44 antibody: Provide supplier name: BioLegend, Cat. # 103044, Clone: IM7.
PE-conjugated anti-mouse CD69 antibody: Provide supplier name: BioLegend, Cat. # 104508, Clone: H1.2F3.
APC-conjugated anti-mouse CD103 antibody: Provide supplier name: BioLegend, Cat. # 121414, Clone: 2E7.
PE/Cyanine7-conjugated anti-mouse CD11c antibody: Provide supplier name: BioLegend, Cat. #117318, clone: N418.
APC-conjugated anti-mouse CD4 antibody: Provide supplier name: BioLegend, Cat. #100412, Clone: GK1.5.
PE-conjugated anti-mouse IFN-gama antibody: Provide supplier name: BioLegend, Cat. #505808, Clone: XMG1.2.
Brilliant Violet 421 conjugated anti-mouse TNF-α antibody: Provide supplier name: BioLegend, Cat. # 506328, Clone: MP6-XT22.
FITC-conjugated anti-mouse CD4 antibody: Provide supplier name: BioLegend, Cat. #100406, Clone: GK1.5.
PE-OVA323-339 tetramer antibody: Provide supplier name: MBL, Cat. TS-M710-1.
Brilliant Violet 510-conjugated anti-mouse CD8a antibody: Provide supplier name: BioLegend, Cat. #100804, Clone: 5H10-1.
PE-OVA257-264 tetramer antibody: Provide supplier name: MBL, Cat.TS-5001-1C.
Brilliant Violet 421-conjugated anti-mouse CD44 antibody: Provide supplier name: BioLegend, Cat. # 103044, Clone: IM7.
APC-conjugated anti-mouse IgG antibody: Provide supplier name: BioLegend, Cat. #405308, Clone: Poly4053.
PerCP/Cyanine5.5-conjugated anti-mouse B220 antibody: Provide supplier name: BioLegend, Cat. #152406, Clone: 1D3/CD19.
Brilliant Violet 510 anti-mouse IgD antibody: Provide supplier name: BioLegend, Cat. #405723, Clone: 11-26c, 2a.
Brilliant Violet 421 anti-mouse CD138 antibody: Provide supplier name: BioLegend, Cat. #142508, Clone: 281-2.
PE/Cyanine7 anti-mouse CD38 antibody: Provide supplier name: BioLegend, Cat. #102718, Clone: 90.
APC-conjugated anti-mouse IL-4 antibody: Provide supplier name: BioLegend, Cat. #504105, Clone: 11B11.
PE/Cyanine7-conjugated anti-mouse IFN-gama antibody: Provide supplier name: BioLegend, Cat. #505808, Clone: XMG1.2.
PE-conjugated anti-human-IgG Fc antibody: Provide supplier name: BioLegend, Cat:410708, Clone: M1310G05.

The following antibodies were used for ELISA:
Goat anti-mouse IgG (1:10000, Cat.No. 1030-05), IgG1 (1:10000, Cat.No. 1070-05), IgG2a (1:10000, Cat.No. 1080-05), IgG2b (1:10000, Cat.No. 1090-05), IgG2c (1:10000, Cat.No. 1079-05), IgG3 (1:10000, Cat.No. 1100-05), IgA (1:5000, Cat.No. 1040-05), and HRP-conjugated goat anti-rabbit IgG (1:5000, Cat.No. 4030-05) were purchased from Southern Biotech.

The following antibody were used for ELISpot assay
HRP-conjugated goat anti-mouse IgG (1:10000, Cat. #SA5-10276) secondary antibody (Invitrogen, USA)

The following antibodies were used for immunofluorescence staining
Human anti-S primary antibody: Provide supplier name: Sino Biological Inc, Cat: 40150-001; 1:200 dilution.
F488-anti-human secondary antibody: Provide supplier name: Proteintech, Cat: SA00003-12; 1:200 dilution.
SARS-CoV-2 Spike antibody, Rabbit PAb, Provide supplier name: Sino Biological Inc, Cat: 40589-T62, 1:1000 dilution
Goat-anti rabbit IgG secondary antibody, Provide supplier name: Invitrogen, Cat: A11008, 1:1000 dilution

The following antibody were used to block MHC II

	Neutralizing anti-mouse I-A/I-E antibody (1:200, Cat. #107602, BioLegend)? Rat IgG2b, κ (1:200, Cat. #400601, BioLegend)
Validation	All the antibodies were freshly purchased from the companies that provided certifcates of validation analysis.

Eukaryotic cell lines

Policy information about cell lines and Sex and Gender in Research

Cell line source(s)	Sf9 insect cells (provided by our laboratory) , ACE2-expressing HEK293T cells (293T/ACE2, constructed by our group, a mouse NEC cell line (BLUEFBIO, CAT: BFN6021547), Vero E6 cells (provided by the Chinese Academy of Medical Sciences and Peking Union Medical College).
Authentication	No authentication has been used.
Mycoplasma contamination	Neither of the cell lines used in this study tested positive for Mycoplasma.
Commonly misidentified lines (See ICLAC register)	No commonly misidentified lines were used.

Animals and other research organisms

Policy information about studies involving animals; ARRIVE guidelines recommended for reporting animal research, and Sex and Gender in Research

Laboratory animals	Specific pathogen-free (SPF) BALB/c and C57BL/6 mice (5-7 weeks, wild-type) were purchased from HFK bioscience company (China) and maintained at 22–23°C, 45–55% relative humidity with a 12-12 h light-dark cycle with free food and water supplies. New Zealand rabbits were purchased from Pizhou Oriental Group.
Wild animals	No wild animals were used in this study.
Reporting on sex	Findings do not apply to one sex. Female animals were chosen because males are aggressive and often fight and get injured, which interferes with data collection.
Field-collected samples	No field-collected samples were used in the study.
Ethics oversight	SARS-CoV-2 viral challenge experiments were performed in the ABSL-4 facility of Kunming National High-level Biosafety Primate Research Center with approval from the Institutional Animal Care and Use Committee of the Institute of Medical Biology, Chinese Academy of Medical Sciences. All the other animal experiments in this study were performed in accordance with the guidelines approved by the Animal Care and Use Committee of Sichuan University (Chengdu, Sichuan, China).

Note that full information on the approval of the study protocol must also be provided in the manuscript.

Flow Cytometry

Plots

Confirm that:

☒ The axis labels state the marker and fluorochrome used (e.g. CD4-FITC).

☒ The axis scales are clearly visible. Include numbers along axes only for bottom left plot of group (a 'group' is an analysis of identical markers).

☒ All plots are contour plots with outliers or pseudocolor plots.

☒ A numerical value for number of cells or percentage (with statistics) is provided.

Methodology

Sample preparation	Cells in BAL<br>BAL was obtained by injecting 0.5 mL ice PBS twice. Cells from BAL were harvested by centrifugation (1500 rpm, 3 min), resuspended with RPMI 1640 medium, seeded into 12-well plates, and restimulated with peptide pools for RBD for 16 h including 4 h treatment with BFA for the analysis of T cell responses.<br><br>Cells in lung<br>Intranasally vaccinated BALB/c or C57BL/6 mice were sacrificed on day 7 and 28 after the third immunization, respectively. Lung tissues were first minced into extremely small pieces and digested in DMEM containing collagenase type 1 (0.5%) and collagenase type ? (0.5%) in a 37 °C incubator for one hour. The digested lung tissues were filtered with 70 mesh cell strainers, treated with red blood cell lysis buffer, and washed three times to obtain single-cell suspensions for FCM detection. Some single cells from lung tissues were restimulated with RBD peptide pools or irrelevant antigen for 16 h as mentioned before for FCM analysis.

B cells in spleen, bone marrow and blood
Spleen, bone marrow and blood were collected, lysed, washed twice, and stained with antibodies to determine RBD-specific IgG-producing plasma cells and MBCs. Cells were stained with APC-conjugated anti-mouse IgG (Cat. #405317; Clone: Poly4053), PerCP/Cyanine5.5-conjugated anti-mouse CDB220 (Cat. #152406; Clone: 1D3/CD19), Pacific Blue anti-mouse IgD (Cat. #405712; Clone: 11-26c, 2a), and Brilliant Violet 421 anti-mouse CD138 (Cat. #142508; Clone: 281-2). PE/Cyanine7 anti-mouse CD38 (Cat. #102718; Clone: 90) and FITC-conjugated anti-mouse CD80 (Cat. #104706; Clone: 16-10A1).

Splenocytes activation
Lymphocytes from spleen and i lymph nodes were isolated to investigate T cellular immune responses. lymphocytes from spleen were isolated at a sterile condition and cultured in complete 1640 medium supplied with 10% FBS, 100 μg/ml streptomycin, 100 U/ml penicillin, 1 mM pyruvate (all from Gibco, USA), 50 μM β-mercaptoethanol, and 20 U/ml IL-2 (all from Sigma-Aldrich, USA). 10 μg/ml RBD was added to activate cells. Before cell staining, brefeldin A (BFA, BD Biosciences) were used to block intracellular cytokine secretion. Culture supernatants were collected to measure the levels of IL-4 and IFN-γ by ELISA. Cells were stained with PerCP/Cyanine5.5-conjugated anti-mouse CD3 (Cat. #100218; clone: 17A2), APC-conjugated anti-mouse CD4 (Cat. #100412; Clone: GK1.5), FITC-conjugated anti-mouse CD8a (Cat. #100804, Clone: 5H10-1), Brilliant Violet 421-conjugated anti-mouse CD44 (Cat. # 103044, Clone: IM7) and PE-conjugated anti-mouse CD69 (Cat. #104508; Clone: H1.2F3). Functional T cells in the spleen were stained with the following antibodies: PerCP/Cyanine 5.5-conjugated anti-mouse CD3 (Cat. 100218; Clone: 17A2), PE-conjugated anti-mouse CD4 (Cat. #100206; Clone: 17A2), FITC-conjugated anti-mouse CD8a (Cat. #100804, Clone: 5H10-1), APC-conjugated anti-mouse IL-4 (Cat. #504105; Clone: 11B11) and PE/Cyanine7-conjugated anti-mouse IFN-gama (Cat. #505808; Clone: XMG1.2).

Blockade of RBD binding to ACE2 receptor
In the experiment blocking the binding of RBD to ACE2, we used three RBD-Fc proteins, RBD-WT, RBD-Delta, and RBD-Omicron. The 35-day sera of each group were added to 0.3 μg/mL RBD-Fc protein in BPBS (0.1% BSA in PBS) at a 1:270 dilution and incubated for 30 min at room temperature. RBD-Fc with or without sera was subsequently added to 293T/ACE2 cells and incubated for 30 min at 4 ?.

Instrument

Flow cytometric data were collected using NovoCyte (ACEA bioscience. Inc).

Software

NovoExpress 1.4.1.

Cell population abundance

We performed the flow cytometry for phenotypes of the T cells with the production of IFN-γ, IL-4 or expression CD44, CD69 by analysing the stimulated lymphocytes in spleen and lung, and is easy to get enough CD8+ or CD4+ cell for further gating cells.

We performed the flow cytometry for phenotypes of the T cells with the expression CD44, CD103 and CD69 by analysing the lymphocytes in lung, and is easy to get enough CD8+ or CD4+ cell for further gating cells.

We performed the flow cytometry for phenotypes of the RBD-specific GCB, memory B cells and plasma cells with the expression IgG and CD80 and CD69 by analysing the lymphocytes in spleen, bone marrow and blood, and is easy to get enough CD138+ or CD38+ cell for further gating cells.

Gating strategy

Plasma cells were gated from IgD-, B220- and CD138+, and RBD+, and IgG+ plasma cells were define as RBD-specific IgG-producing plasma cells.

Memory B cells were gated from IgD-,CD138-, B220+, CD38+ and CD80+, and RBD+ memory B cells were define as RBD-specific memory B cells.

T Cells in spleen were gated from CD3+, CD4+ or CD8+ cells . From these gated cells the percentage of CD69, CD44, IFN-gamma and IL-4 positive cells were recorded.

B220--, CD4+, PD-1+ and CXCR5+ cells were define as Tfh. GCB were gated from CD3-, CD19+, CD95+ and GL-7+, and RBD +GCB were define as RBD- specific GCB.

T Cells in BAL or lung were gated from CD3+, CD4+ or CD8+ cells, and IFN-γ+ CD4+ or CD8+ cells were define as antigen specific CD4 or CD8 T cells.

T Cells in BAL or lung were gated from CD3+, CD4+ (CD8+) and CD44+ cells. CD69+ and CD103+ T cells were define as antigen experienced TRMs.

ACE2 transfected -Cells with PE fluorescence intensity were counted for the inhibition of receptor binding assays.

☒ Tick this box to confirm that a figure exemplifying the gating strategy is provided in the Supplementary Information.
